# Just-in-time adaptive ecological momentary assessment (JITA-EMA)

**DOI:** 10.3758/s13428-023-02083-8

**Published:** 2023-02-25

**Authors:** Stefan Schneider, Doerte U. Junghaenel, Joshua M. Smyth, Cheng K Fred Wen, Arthur A. Stone

**Affiliations:** 1 Dornsife Center for Self-Report Science & Center for Economic and Social Research, University of Southern California, 635 Downey Way, Los Angeles, CA 90089-3332 USA; 2 Department of Psychology, University of Southern California, Los Angeles, CA USA; 3 Leonard Davis School of Gerontology, University of Southern California, Los Angeles, CA USA; 4 Biobehavioral Health and Medicine, Pennsylvania State University, State College, PA USA

**Keywords:** Ecological momentary assessment, Just-in-time adaptive intervention, Computerized adaptive testing, mHealth

## Abstract

Interest in just-in-time adaptive interventions (JITAI) has rapidly increased in recent years. One core challenge for JITAI is the efficient and precise measurement of tailoring variables that are used to inform the timing of momentary intervention delivery. Ecological momentary assessment (EMA) is often used for this purpose, even though EMA in its traditional form was not designed specifically to facilitate momentary interventions. In this article, we introduce just-in-time adaptive EMA (JITA-EMA) as a strategy to reduce participant response burden and decrease measurement error when EMA is used as a tailoring variable in JITAI. JITA-EMA builds on computerized adaptive testing methods developed for purposes of classification (computerized classification testing, CCT), and applies them to the classification of momentary states within individuals. The goal of JITA-EMA is to administer a small and informative selection of EMA questions needed to accurately classify an individual’s current state at each measurement occasion. After illustrating the basic components of JITA-EMA (adaptively choosing the initial and subsequent items to administer, adaptively stopping item administration, accommodating dynamically tailored classification cutoffs), we present two simulation studies that explored the performance of JITA-EMA, using the example of momentary fatigue states. Compared with conventional EMA item selection methods that administered a fixed set of questions at each moment, JITA-EMA yielded more accurate momentary classification with fewer questions administered. Our results suggest that JITA-EMA has the potential to enhance some approaches to mobile health interventions by facilitating efficient and precise identification of momentary states that may inform intervention tailoring.

## Introduction

Interest in the use of mobile Health (mHealth) technologies has risen substantially among behavioral researchers and healthcare practitioners in recent years. Mobile devices utilized for mHealth applications (including smartphones, tablets, and ambulatory monitoring devices such as wearable activity trackers) are sophisticated, user-friendly, and affordable. This has created many opportunities for providing healthcare in difficult-to-reach and medically underserved areas and for the delivery of behavioral, psychological, and medical interventions in people’s everyday environments (Kaplan & Stone, 2013; Nahum-Shani et al., 2015). For example, mHealth tools have been used to send reminders to improve medication adherence (Ben-Zeev et al., 2014), to encourage exercise and physical activity (Van Dantzig et al., 2013), and to deliver brief stress management interventions in daily life (Loo Gee et al., 2016).

Arguably one of the biggest promises of mHealth interventions is that they could be designed to deliver treatments at precisely those moments in time when patients are most in need or may most benefit from them. Such intervention approaches have been termed Ecological Momentary Interventions (Heron & Smyth, 2010; Loo Gee et al., 2016; Versluis et al., 2016) or just-in-time adaptive interventions (JITAI; Collins et al., 2004; Nahum-Shani et al., 2015; Nahum-Shani et al., 2018). Although the idea of tailored treatment has a long history, the continuous tracking of individuals in their daily lives with mHealth technologies provides unprecedented opportunities for tailoring the timing and contents of treatments in real time to the occurrence of presumptive risk states or other “opportune moments” for each individual. For instance, for interventions promoting healthy eating habits, time periods of elevated stress or fatigue can be critical for dietary lapses (Forman et al., 2019), and delivering interventions when a person’s stress or fatigue experiences reach a certain predefined threshold value could potentially be most effective for influencing a person’s health behavior. Moreover, this threshold may well differ from patient to patient and may vary over time, and it might be ideal for the delivery of treatments to adapt accordingly.

Despite the growing recognition of the rich potential of JITAI for mHealth applications, the development and implementation of these interventions is still in its early stages and requires continued multidisciplinary efforts and advances in dynamic behavior theories (Spruijt-Metz & Nilsen, 2014), study design (Klasnja et al., 2015), intervention techniques (Nahum-Shani et al., 2015), and measurement methods (Collins et al., 2004). One core challenge for JITAI is the measurement of *tailoring variables* that are used to inform the timing, content, and dosage of interventions for each individual (Nahum-Shani et al., 2018). We acknowledge that there are many open questions about how to identify effective tailoring variables that successfully moderate the impact of an intervention. In practice, JITAIs are often designed to deliver interventions shortly before, during, or after a presumptive state of risk or opportunity for a given individual (Hardeman et al., 2019; Perski et al., 2022).

Ecological momentary assessment (EMA, also called experience sampling) methods are indispensable for the measurement of these states, and can be combined with information from wearable devices that can measure behavioral, physiological, or environmental variables in real time using passive sensors (Nahum-Shani et al., 2018). Using EMA, patients typically report their current or recent subjective states and experiences multiple times per day on custom-programmed mobile electronic devices as they go about their normal daily lives (Shiffman et al., 2008). The granular data from EMA is integral to the delivery of JITAI because it captures short-term, within-person processes that cannot be assessed with global retrospective self-reports, and because it can address a wide range of subjective experiences that cannot be measured with passive sensor technologies (Wenze & Miller, 2010). For example, if a JITAI is to be triggered by moments in which patients experience high levels of stress, fatigue, or negative mood, EMA self-reports are routinely used to acquire the necessary information.

Despite the unique opportunities provided by EMA, there are significant barriers to the successful and optimal integration of EMA in JITAI research and practice. EMA is widely employed in observational research, but the measurement technique in its traditional form was not designed specifically to facilitate decisions about the timing of momentary intervention delivery. In contrast to “pull” interventions that make intervention contents available to patients at their disposal, JITAI uses a “push” approach by attempting to select the most appropriate timing and content of interventions for each individual. Next to a host of EMA design considerations (e.g., the temporal density of assessments, which contents to measure), this places particularly high demands on psychometrically sound EMA measurement within individuals.

One of the challenges to the seamless integration of EMA into JITAI is the need to keep participant burden low while achieving high measurement precision. To be suitable for use as tailoring variables, momentary assessments are required to have very little measurement error. Common EMA measures such as momentary emotion scales with 3–5 items have been shown to capture within-person fluctuations with reliabilities of 0.6 to 0.8 (Cranford et al., 2006; Scott et al., 2018). Whereas this level of reliability is often considered sufficient to detect group-based relationships in observational research, as noted by Nunnally (1978), higher standards apply where decisions are made for each individual on the basis of specific test scores. Even when reliability is as high as 0.9, the standard errors of measurement of observed scores are nearly one-third as large as the standard deviation of the scores themselves. Imprecision due to measurement error in EMA directly translates into inaccurate (false positive and false negative) decisions about treatment delivery in JITAI: the more a measurement is impacted by random error, the more will the decisions about when to deliver a treatment themselves be random rather than tailored to the individual’s changing states (Collins et al., 2004).

An obvious strategy to increase measurement precision (i.e., reduce random error) is to increase the number of items in a given EMA scale (Calamia, 2019). However, a challenge associated with JITAI is participant burnout, that is, the risk that individuals lose motivation to engage in the intervention over time or that they abandon the intervention altogether (Nahum-Shani et al., 2018). Keeping EMA surveys very brief is essential to limit response burden (Intille et al., 2016). Long EMA surveys have been related to lower participant compliance in observational studies (Eisele et al., 2020), and may contribute to intervention fatigue and participant burnout in JITAI.

These two aspects of EMA measurement, the need for brief EMA surveys while achieving high levels of measurement precision, tug against each other and often require interventionists to make dissatisfying compromises when choosing or designing EMA surveys for use in JITAIs. However, it is also the case that conventional EMA administration methods are not optimized to address the needs of JITAI. Whereas JITAIs are designed to adapt to the individual’s changing states, conventional EMA administration is inflexible in the sense that the same set of questions are administered to respondents regardless of their current state. We propose that a strategy where the specific questions administered in EMA adapt to the individual’s changing states would be more efficient when used to make treatment decisions in JITAIs.

Accordingly, we propose *just-in-time adaptive EMA* (JITA-EMA) as an assessment technique to increase the efficiency of EMA; we believe this technique may have particular value when used as a tailoring variable in JITAI. The goal of JITA-EMA is to administer the *smallest number* and *optimal selection* of momentary questions necessary to enable the classification of momentary states with high accuracy at each measurement occasion. To accomplish this, JITA-EMA builds on computerized adaptive testing (CAT) methods developed for purposes of classification (also called computerized classification testing, CCT) (Thompson, 2007). These methods have been successfully implemented to make categorical (“pass vs. fail,” or “treat vs. do not treat”) decisions in educational settings for many years. Thus, a framework for many components of JITA-EMA is already available, even if not yet incorporated into mHealth settings involving data collection with EMA (Gibbons, 2017; Rose et al., 2012). Specifically, whereas CCT has been developed mainly for between-person decisions (e.g., who meets a given criterion to receive a treatment), we propose JITA-EMA as a dynamic extension of CCT to classify momentary states that may inform JIT treatment implementation.

We emphasize that even though measurement error sets an upper limit for the precision of intervention tailoring decisions, psychometrically sound and efficient EMA measurement by itself does not ensure more effective tailoring of interventions. For example, one important gap in the development of optimal JITAIs in many areas is a shortage of empirical evidence about which constructs adequately capture a state of momentary risk or opportunity for change or predict a pre-specified proximal outcome (e.g., smoking in the next few minutes, or increased symptom severity on the next day) (Nahum-Shani et al., 2015). If the constructs used as tailoring variables in an intervention are not related to a relevant proximal outcome, then no level of EMA measurement precision will help with effective intervention tailoring. The purpose of the proposed psychometric approach is to develop techniques for the assessment of momentary self-reported experiences to be more closely aligned with the goals of adaptive intervention delivery by achieving accurate classification of momentary states with little participant burden, as one of many strategies to facilitate improved treatment tailoring decisions in JITAI.

In the remainder of the paper, we first present the basic components of JITA-EMA. We then present results from two simulation studies to explore the potential usefulness of the method for more efficient classification of momentary states based on EMA. We end with a discussion about potential opportunities and challenges when using JITA-EMA in JITAI research and practice.

## Components of JITA-EMA

**Fig. 1:**
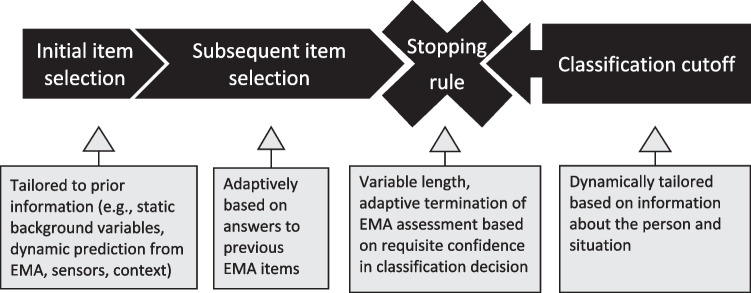
Components of just-in-time adaptive ecological momentary assessments (JITA-EMA)

The proposed building blocks of JITA-EMA are schematically illustrated in Fig. 1. Treatment decisions in JITAI are most commonly based on “if-then” rules, which control that an intervention is offered only if a (binary) criterion is met (e.g., *if* current fatigue level > cutoff *then* deliver a momentary intervention) (Nahum-Shani et al., 2018). Accordingly, JITA-EMA should both efficiently quantify an individual’s momentary states and efficiently classify each momentary state with respect to a dynamically changing criterion state. Heuristically, we propose that this can be achieved following principles of effective interpersonal communication, that is, by administering EMA questions in ways that resemble a goal-directed transactional process (Hargie, 2011):

1. *Initial item selection*: Rather than treating the individual as a “blank slate,” JITA-EMA starts with an EMA question that is tailored to prior knowledge about the person’s internal states in similar situations.
2. *Subsequent item selection*: Rather than asking a predetermined set of questions, JITA-EMA strategically administers EMA questions tailored to a person’s answers to previous questions.
3. *Stopping rule*: Rather than asking a fixed number of questions, JITA-EMA stops administering EMA questions as soon as, and no later than, the individual’s current state has been classified with requisite confidence.
4. *Classification cutoff*: Rather than applying the same standards to all people at all times, JITA-EMA allows the tailoring of cutoffs used for classification based on prior information about the person and situation.

### Psychometric underpinnings of JITA-EMA

The components of JITA-EMA are rooted in item response theory (IRT), which forms the basis for CAT and CCT. IRT methods have a long history in educational testing and have more recently been successfully adopted for patient-reported outcome measurement as implemented in PROMIS® (Cella et al., 2010), Neuro-QoL (Gershon et al., 2012), or SCI-QOL (Tulsky & Kisala, 2015). To date, patient-reported outcomes with IRT have almost exclusively focused on traditional between-person measurement contexts, but IRT methods are applicable to within-person measurement with EMA (Fayers, 2007; Rose et al., 2012; Wainer, 2000). The cornerstone of IRT measurement is an item bank: a collection of questions that represent a well-defined, typically unidimensional construct (e.g., fatigue, negative affect). Much like confirmatory factor analysis, IRT represents the construct of interest as a latent variable, called “theta,” that can be estimated from responses to these questions. The probability of a person choosing a given response on any item in the bank is a mathematical function of the person’s state (their “theta” level) and specific parameters of the items (threshold and discrimination parameters) (Fayers, 2007).

Two aspects of IRT are critical for item administration via CAT. First, when the parameters of the items in a bank are known (because the items have been rigorously tested to behave in the same way across groups and individuals, and have been normed in a suitable sample), any combination or subset of items from the bank produces scores on the same metric, regardless of how many and which items are administered. This sets the stage for strategic item selection. Second, IRT estimates information functions that explicitly acknowledge that some items are more informative (i.e., more reliable, with less measurement error) at higher theta levels, whereas other items are more informative at lower theta levels (unlike classical test theory, where an item is assumed to have the same measurement precision at all levels). This opens the door for CAT and CCT algorithms, in which those items are consecutively selected that are deemed most informative given the current “best guess” about the individual’s true theta level, while items that are presumably less relevant are omitted (Wainer, 2000; Weiss, 2004; Weiss & Kingsbury, 1984). As described next, JITA-EMA builds on these basic mechanisms that are akin to all CAT and CCT assessments and adapts them to provide efficient assessment of dynamically changing states within individuals.

#### Using prior information for initial item selection

Every CAT starts off with a “best guess” about the person’s theta level in order to decide which item from the item bank should be administered first (Weiss & Kingsbury, 1984). In traditional (e.g., cross-sectional) measurement settings, where little to nothing is generally known about an individual at the outset of an assessment, CATs are often initialized using an item that is most informative for the “average” person, such that the first item is not tailored to the individual or situation (Choi et al., 2010) (even though procedures for tailored CAT initialization based on “collateral” information about a person exist; van der Linden, 1999). In contrast to settings that involve assessment of an individual at a single time point, JITA-EMA requires that the CAT be initialized repeatedly, that is, at the beginning of each EMA prompt. This simultaneously renders the choice of the initial item more important and provides many more opportunities for tailoring the selection of the first item to the individual and current situation.

Although little may be known about a person at the beginning of a JITAI period, this changes quickly as intensive longitudinal (EMA) assessments accumulate. After a few EMA prompts have occurred, an informed guess can be developed about each person’s general or average theta level compared to other individuals on the construct of interest. As more momentary assessments accumulate over time, it becomes possible to derive estimates of person-by-situation interactions which can be used to guess an individual’s typical state within the current situation. For example, to initialize a momentary fatigue assessment, JITA-EMA could capitalize on the fact that fatigue has a typical diurnal cycle (which may differ from person to person) (Stone et al., 2006), and initialize a CAT using an item that best reflects the predicted fatigue level of that person at the time of day the assessment takes place, based on their ideographically estimated diurnal cycle. In addition, multiple sources of information (e.g., momentary reports of fatigue, affect, activities) can be taken into account for initial item selection when analytically combined (e.g., using multivariate time series models; van der Krieke et al., 2015).

#### Adaptive selection of subsequent items

After a respondent answers the first item administered in a given assessment session (or EMA prompt), a CAT algorithm reevaluates the person’s most likely theta level and applies the presumably most informative item for the current “interim theta” estimate (Choi et al., 2010; Fayers, 2007). This process is repeated for each subsequent item administered. At this stage of the assessment, JITA-EMA is no different from CAT administration in other research or practical settings. However, due to the high participant burden associated with EMA, and the desire for “ultra-brief” measurements with no more than 2–5 items (or even a single item) per construct administered at each prompt, there is a greater urgency for stopping the administration of items (i.e., to estimate a “final theta” value for the person’s current state) adaptively and as soon as reasonably possible.

#### Adaptive stopping rules

CAT algorithms can be programmed with different “stopping rules” that tell the program when to end an assessment and to record the final theta value (Wainer, 2000; Weiss, 2004; Weiss & Kingsbury, 1984). The simplest rule is to stop after a predefined total number of items have been administered. This “fixed” stopping rule is the same as what is implicitly applied when nonadaptive assessment methods are used. A second, “variable” rule is to stop administering items whenever the person’s theta level is estimated with a predefined level of measurement precision (a sufficiently small measurement error or confidence interval). This second stopping rule is attractive in many observational settings, because it attempts to attain a uniformly high measurement precision across the full range of possible theta values (e.g., across all possible fatigue levels).

When the goal is to classify states for sufficiently accurate treatment tailoring decisions as in JITA, however, a uniformly high measurement precision may not be required. For JITA-EMA to be most efficient, we recommend an alternative “variable” stopping rule that is commonly applied in CCT. This stopping rule administers items until a classification decision is made with sufficient (e.g., 95%) confidence, regardless of the measurement precision of the observed theta value (Thompson, 2007). The idea is that high measurement precision (low measurement error) of the theta value is needed if a person’s current theta level is close to the cutoff score (slightly above, or slightly below the threshold); in this case, administering more items is desirable to achieve accurate classification. On the other hand, lower measurement precision (with higher measurement error of the theta value) is entirely tolerable if a person’s theta level is estimated to fall into ranges that are far from (very much above or below) the classification threshold; in this case, administering only very few items may suffice to meet the goal of classifying a person’s current state with high confidence.

We note that stopping rules in CCT generally use symmetric confidence bounds (e.g., a classification decision could be made when the classification cutoff is either below the 2.5th percentile or above the 97.5th percentile of the estimate), such that false positive and false negative classification decisions tend to be given equal weight. Depending on the type of intervention to be provided, this may not be desirable in all instances. An interventionist may deem the potential benefits of identifying true positives as high and the costs associated with false positives as low, for example, when a positive classification decision triggers access to a support hotline which patients who currently do not need the support can readily dismiss. For another intervention, identifying true negatives may be especially important and the potential costs of false positives may be deemed high, for example, when there is a high risk that patients will get annoyed or frustrated when interventions are repeatedly offered at inopportune or inconvenient times. Specific needs to avoid false positive or false negative classification decisions could be taken into consideration when applying variable stopping rules in CCT.

#### Dynamically tailoring the classification cutoffs

CCTs employed in educational testing typically hold the cutoffs for classification constant across individuals to ensure equal standards for all individuals. Such uniform cutoffs, albeit not tailored to the individual, are also commonly applied in JITAIs (see Perski et al., 2022). For example, at each EMA prompt, a momentary fatigue level of one standard deviation above the population mean could be classified as “elevated” fatigue, which could be used to trigger a momentary intervention. However, a core feature of JITAI is that the decisions about when to intervene adapt to the dynamically changing states of each individual (Nahum-Shani et al., 2018). Although not a genuine component of CAT in other areas, prior information in EMA applications can be directly incorporated in CAT algorithms to dynamically tailor the cutoffs for the classification of current states (Chalmers, 2016). For example, a dynamically tailored cutoff in JITA-EMA could be defined as a momentary fatigue level that is higher than usual for the individual and present time of day, given the fatigue diurnal cycle estimated from the previous EMA prompts for that individual. More complex tailored cutoffs could also be developed, such as cutoffs considering specific changes within the individual (including short-term shifts, changes within a predefined moving time window, or cumulatively over the course of a study) (see Smyth et al., in press). We note that even though it is possible to apply dynamically tailored cutoffs in CAT, there are many intricate decisions about how to operationally define appropriate cutoffs for a given application and how to analytically derive them. Addressing these questions is beyond the scope of this paper.

It is also important to note that dynamically tailored classification cutoffs that are empirically derived from a person’s data contribute additional measurement error to classification decisions. In the fatigue example, a person’s diurnal cycle will be estimated with uncertainty (i.e., with sampling variance), and this source of error will be more pronounced for individuals with diurnal cycles that are less consistent and more variable from day to day. Consequently, decisions about treatment delivery in JITAI can be impacted not only by unreliable measurement at each EMA prompt, but also by unreliability in decisions about dynamically tailored cutoffs. This is likely to make efficient yet precise classifications of an individual’s changing states—the purpose of JITA-EMA—all the more important.

## Simulation studies

We conducted two simulation studies to evaluate whether JITA-EMA improves the efficiency and accuracy of momentary classification decisions compared to usual EMA item selection methods. The advantage of a simulation study over an empirical study is that the true momentary states and the true cutoff values for classification are known in a simulation. This makes it possible to directly compare the true classifications with those that would be obtained with different item selection methods. The R package mirtCAT (Chalmers, 2016) was used for the simulations. This versatile package can be used to administer and simulate both adaptive and nonadaptive tests using IRT, and to build graphical user interfaces for administering CATs in real time. R scripts of the simulations can be accessed at https://osf.io/jte5h/.

### Overview of the simulations

Both simulations focus on a representative use-case regarding the assessment of momentary fatigue, a self-reported experience with broad applicability as a tailoring variable in JITAIs (Ben-Zeev et al., 2014; Goldstein et al., 2017). Elevated daytime fatigue is a significant health concern and represents a symptom or risk factor for various physical and mental health problems such as depression and anxiety (DeLuca, 2005). It is also generally believed that moments of exacerbated daytime fatigue make individuals temporally more vulnerable to poor self-care behavior and increase the likelihood of minor and major accidents. In this use-case scenario, we assume that moments of elevated daytime fatigue represent a potential tailoring variable for a momentary intervention timing decision. The specific decision made is outside of the purview of JITA-EMA and accordingly, the simulations are agnostic to the treatment decision (e.g., high daytime fatigue could be viewed as a risk state that may trigger an intervention, or it could be viewed as a state of low receptivity where intervention delivery should be withheld).

The two simulations should be viewed as examples to illustrate the various components of JITA-EMA as an item selection method, not as a prescription for an ideal implementation in applied settings. Our first simulation (study 1) examines a hypothetical setting in which a researcher adopts a *uniform* cutoff for the classification of momentary fatigue states. This simulation does not consider dynamic cutoffs and the goals of the simulation were to evaluate the performance of measurement aspects that are genuine to CCT: the potential benefits of adaptive stopping rules over fixed-length EMA assessments and the benefits of adaptive over nonadaptive (initial and subsequent) item selection. The cutoff was held the same for each person and situation, where we assume that a researcher is interested in the detection of momentary fatigue at a level that is likely debilitating for most individuals. Correspondingly, momentary fatigue was considered above the cutoff if the observed score exceeded a *z*-score of 1 (i.e., approximately the 85th percentile of scores) on a fatigue measure that we assume has been previously calibrated and normed using IRT.

The second simulation (study 2) examines a more complex situation that incorporates all of the proposed JITA-EMA components and where treatment decisions are based on *dynamically tailored* (by individual and time of day) classification cutoffs. Here, we assume that a researcher is interested in the detection of elevated daytime fatigue, using cutoffs that are tailored to a person’s idiographic diurnal cycle of fatigue. The fatigue diurnal cycles were estimated from the fatigue theta scores that had been previously observed for a given person up to the person’s current moment in the study. Elevated daytime fatigue was defined as fatigue levels between 6 AM and 6 PM (considered daytime) that are at least half a (within-person) standard deviation higher than expected *for the individual at a given time of day* (half a standard deviation was chosen because it is considered a benchmark for minimally important differences for many symptoms and experiences, see Norman et al., 2003).

### Generation of “true” fatigue values

**Fig. 2:**
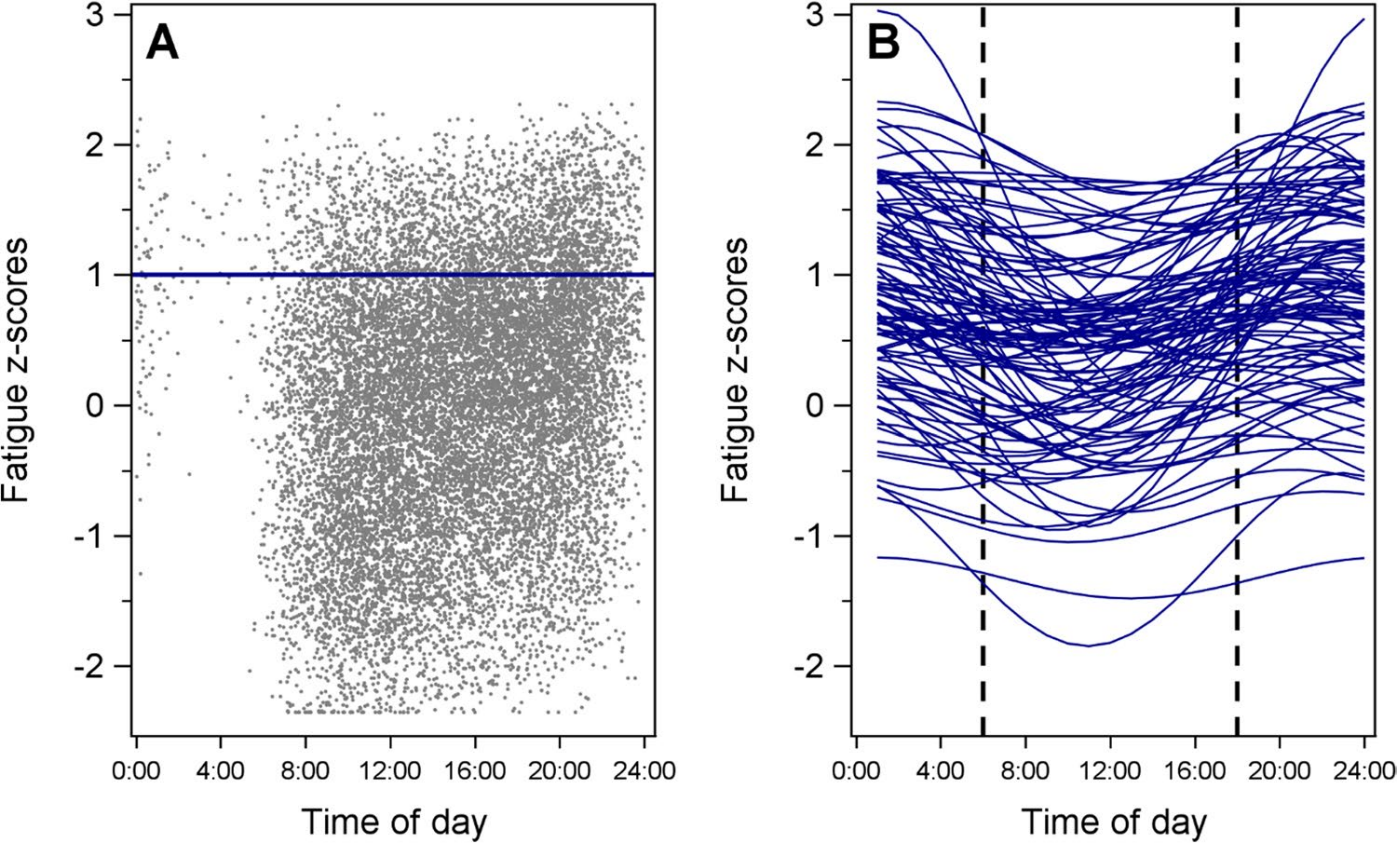
True fatigue values and true classification cutoffs for the simulations. **A** Gray dots represent the true momentary fatigue values for both simulation studies; the solid horizontal line indicates the uniform classification cutoff for study 1. **B** Solid horizontal lines indicate the dynamic classification cutoffs for study 2, where each solid line is for an individual; dashed vertical lines reference the time period from 6 AM to 6 PM used for classification in study 2

Our aim was to evaluate the accuracy and efficiency of momentary classifications a researcher would have obtained with different methods for EMA item selection using a real-world example. For this reason, rather than generating true momentary fatigue values from a hypothetical population (as would be done in a Monte Carlo simulation), we used momentary fatigue data from a real data set to represent the “true” fatigue states, that is, we assume that these were measured without error. The fatigue values were derived from a pre-existing EMA data set of 106 patients with rheumatoid chronic pain, where momentary fatigue data had been collected five to six times per day for up to 28 days (for details, see Broderick et al., 2009; Broderick et al., 2008). Compliance with the EMA protocol was high, with patients completing on average 91% of the EMA prompts (Broderick et al., 2009). This provided a total of 16,587 “true” momentary fatigue values from the 106 patients (see Fig. 2A). We standardized (*z*-scored) the momentary fatigue values (based on the sample’s overall mean and variance) to place them on a metric that is most common in IRT (mean = 0, *SD* = 1 for theta values in IRT).

### Generation of “true” classification cutoffs

For study 1, the classification cutoff was set at 1 *SD* above the average fatigue level (a *z*-score of 1.0, shown in Fig. 2A).

For study 2, because the simulation included dynamic classification cutoffs that would have been empirically estimated for each individual up to a given point in the study, it was necessary to generate “true” fatigue diurnal cycles for each individual to be able to compare the observed and true classifications of momentary fatigue states. The diurnal cycles were derived from the true momentary fatigue values available for each person in the Broderick et al. (2009) EMA data set. Many statistical models to estimate cyclic changes (diurnal cycles) exist (Chow et al., 2007). For the present illustration, we estimated a “cosinor” regression model using the sine and cosine of time (in hours) of day as predictors, separately for each individual. The following regression model was estimated:

1 $${\textrm{theta}}_i={\upbeta}_0+{\upbeta}_1\sin \left(\left[2\uppi /24\right]{\textrm{HOUR}}_i\right)+{\upbeta}_2\cos \left(\left[2\uppi /24\right]{\textrm{HOUR}}_i\right)+{r}_i$$

where theta_*i*_ are the momentary fatigue values, β_0_ is an intercept, β_1_ and β_2_ are the coefficients associated with the sine and cosine of time of day (HOUR), and *r*_*i*_ is a residual term. The true classification cutoffs for each individual and time of day were defined as the model-predicted values +0.5 (residual within-person) *SD*s. As shown in Fig. 2B, individuals differed markedly in levels and temporal patterns of the resulting “true” dynamic cutoffs.

### Momentary fatigue items used in the simulations

As explained above, the implementation of JITA-EMA requires that subsets of items can be selected from a larger item bank. The 13 fatigue items from the Functional Assessment of Chronic Illness Therapy–Fatigue scale (FACIT-F) represent such an item bank and they were used for the present simulations. FACIT-F items have been calibrated using IRT methods (Lai et al., 2003), can be administered using fixed-length or CAT methods, and are included in the widely used PROMIS fatigue instrument (Cella et al., 2010; Lai et al., 2011). All items use a five-point numeric rating scale (*not at all* to *very much*). Items are worded in present tense (e.g., “I feel tired”, “I feel listless [washed out]”), consistent with the wording of many EMA instruments. The mirtCAT package was used to simulate plausible response patterns for the FACIT-F items given the true momentary fatigue theta values and the IRT item parameters documented for the FACIT-F (Lai et al., 2011).

### EMA item selection methods tested

Five different EMA item selection methods were examined. For each method, we used mirtCAT to simulate the administration of selected FACIT-F items and to generate observed fatigue theta scores (i.e., “scale” scores) by individual and EMA prompt.

1. *Fixed length, two items:* In this condition, exactly two fatigue items were administered at each EMA prompt to simulate a very brief EMA assessment strategy. The items were randomly selected from the 13 FACIT-F items at each prompt.^1^
2. *Fixed length, three items:* Exactly three randomly selected FACIT-F items were administered at each EMA prompt.
3. *Fixed length, five items:* Exactly five randomly selected FACIT-F items were administered at each EMA prompt. Five items would arguably be considered a longer assessment strategy for most JITAI applications.
4. *Variable length:* The purpose of this condition was to examine the potential efficiency benefit of using an adaptive stopping rule, but without adaptive item selection. The maximum number of items to be administered was set at 5 (randomly selected from the FACIT-F as for the fixed-length conditions). However, rather than administering a fixed number of items, the EMA assessment was terminated as soon as the 95% confidence interval of the estimated momentary fatigue level did not include the cutoff for classification.
5. *JITA-EMA*: This condition used the same variable length stopping rule as condition 4, but additionally applied adaptive (initial and subsequent) item selection as proposed for JITA-EMA. Item selection was based on a CAT algorithm that adaptively administered those FACIT-F items that were deemed optimal (i.e., that had the greatest potential to minimize the standard error of the estimated fatigue level) using the maximum expected information criterion (Choi & Swartz, 2009). For the selection of the first (initial) item at each EMA prompt, the CAT was initialized using the momentary fatigue theta score from the patient’s previous EMA prompt (study 1), or using an estimate of the person’s typical fatigue level at the present time of day calculated from a cosinor (with sine and cosine of time of day) regression model fitted to all fatigue theta scores that had been previously obtained for that patient up to the current EMA prompt (study 2).

### Observed classification

In each of the five conditions, observed momentary fatigue theta scores were computed at each EMA prompt from the simulated responses to the specific combination of items administered. The theta scores were derived using maximum a posteriori (MAP) estimation in mirtCAT. MAP is a Bayesian scoring procedure that requires a prior distribution and a normal prior (*SD* = 1.0) was used for all conditions. In study 1, the observed fatigue theta scores were classified as above the cutoff if they exceeded the fixed (uniform) value of 1.0, and below the cutoff otherwise. In study 2, the observed theta scores were classified using dynamically tailored cutoffs that were estimated from the information about an individual’s fatigue diurnal cycle that a researcher would have available at a given point of the study. That is, at each EMA prompt, a cosinor regression model was fitted to the observed theta scores obtained at all prompts that would have been administered to that individual since the beginning of the EMA study up to (and excluding) the current EMA prompt; the person’s model-predicted (i.e., expected) fatigue level for the present time of day was recorded together with the residual *SD* of all previous fatigue scores, and the observed fatigue score for the present prompt was classified as above the cutoff if it was 0.5 *SD*s higher than the expected fatigue level. EMA prompts administered on the first 2 of the 28 days were considered a “run-in” period and were not used for classifying decisions, nor were data collected outside of the 6 AM to 6 PM daytime period.^2^ Observed scale scores obtained during these periods served only to estimate an individual’s dynamic cutoff values. For the variable-length EMA and JITA-EMA methods, the stopping rule during these periods was a standard error of less than 0.3 (a common CAT stopping rule, equivalent to a reliability of 0.90; Thissen, 2000).

### Performance criteria and analyses

The purpose of JITA-EMA is to increase the efficiency of EMA in JITAI, whereby an efficient EMA item selection method should allow accurate classification with minimal items. Accordingly, the two performance criteria were the (1) number of items administered per prompt and (2) the accuracy of the observed classifications in each method.

The number of items administered was known a priori for fixed-length EMAs (i.e., exactly two, three, or five items were administered), but not for the variable-length and JITA-EMA methods. For these two methods, multilevel “null” models (no predictors added, with the number of items per prompt serving as dependent variable) with random intercepts were used to estimate the mean, between-person variance, and within-person variance (across prompts) in the number of items administered.

Classification accuracy was evaluated by examining the sensitivity and specificity of each item selection method. Sensitivity measures the proportion of momentary fatigue states correctly identified as above the cutoff among all moments that were truly above the cutoff (true positive rate). Specificity measures the proportion of momentary fatigue values correctly identified as below the cutoff among those that were truly below the cutoff (true negative rate). Analyses of sensitivity and specificity were conducted using logistic multilevel models as appropriate for clustered binary data (with multiple momentary classifications nested in individuals). Specifically, we estimated random intercepts models of observed classifications (as dependent variable) among those moments that were truly above (for sensitivity) or truly below (for specificity) the cutoff (see Genders et al., 2012). In these models, the estimated intercepts represent the logit sensitivity and logit specificity, respectively, and transforming the logits into probabilities yields the median sensitivities and specificities across individuals. To test whether the logit sensitivities and specificities differed between the EMA item selection methods, multivariate logistic multilevel models were used in which the observed classifications for different item selection methods served as multivariate (i.e., correlated) dependent variables.

In addition to sensitivity and specificity, we also examined Cohen’s kappa to obtain an overall summary measure of agreement between the observed and true momentary classifications for each item selection method. Kappa is frequently used to index the quality of binary classifications when comparing a diagnostic test with a gold standard (Feuerman & Miller, 2008). The kappa statistic can be calculated as a function of sensitivity, specificity, and prevalence values (see Feuerman & Miller, 2008; Thompson & Walter, 1988), and we used this property of the statistic to derive kappa directly from the model parameters of the logistic multilevel models.^3^

The multilevel models were estimated in M*plus* version 8.7 (Muthén & Muthén, 2017) using Bayesian parameter estimation with the program’s default noninformative priors. *P*-values below .05 were considered statistically significant.

### Study 1 results

#### Number of items administered

The variable-length and JITA-EMA methods were programmed to administer a maximum of five items per EMA prompt, but both on average stopped when significantly less than three items were administered (see Table 1). The variable-length method administered a mean of 2.58 items per prompt, and JITA-EMA a mean of 2.30 items (the difference in means was significant at *p* < .01). Specifically, the variable-length method stopped after the very first item for 40.5% of the prompts, after two items for 18.4%, three items for 8.9%, four items for 4.9%, and after five items for 27.3%. JITA-EMA stopped after the first item for 52.5% of the prompts, after two items for 15.1%, three items for 4.5%, four items for 4.3%, and five items for 23.6% of the prompts. The number of items administered varied both between persons (*SD* = .87 for variable-length, *SD* = .84 for JITA-EMA) and within persons (*SD* = 1.43 for variable-length, *SD* = 1.45 for JITA-EMA), where the within-person variation was more pronounced and accounted for 73% (variable-length) and 75% (JITA-EMA) of the total variance. This indicates that the number of items administered varied more by moment than by person.

**Table 1:** Mean number of items administered, sensitivity, specificity, and kappa by EMA item selection method in study 1

	Mean number of items per prompt	Sensitivity	Specificity	Kappa
Two items fixed	2.00	.63 [.59;.66] ^a^	.97 [.96;.98] ^a^	.55 [.45;.63] ^a^
Three items fixed	3.00	.71 [.68;.74] ^b^	.98 [.97;.98] ^a^	.61 [.49;.70] ^b^
Five items fixed	5.00	.77 [.74;.80] ^c^	.99 [.98;.99] ^b^	.76 [.67;.81] ^cd^
Variable length	2.58 [2.42; 2.75]	.73 [.69;.77] ^b^	.99 [.98;.99] ^b^	.72 [.62;.78] ^c^
JITA-EMA	2.30 [2.13; 2.46]	.81 [.78;.83] ^d^	.99 [.98;.99] ^b^	.78 [.68;.83] ^d^

*Note*. Values in squared brackets are 95% credible intervals. Sensitivity, specificity, and kappa values with different superscripts are significantly different between EMA item selection methods at *p* < .05

#### Classification accuracy

As shown in Table 1, for the different fixed-length methods, administering more items per prompt was associated with greater classification accuracy. Median sensitivities significantly increased from 63% (2 items) to 71% (3 items) and to 77% (5 items), specificities significantly increased from 97% (2 items) to 98% (5 items),^4^ and kappa significantly increased from .55 (2 items) to .76 (5 items fixed-length). The variable-length method yielded 73% specificity, 99% specificity, and kappa = .72, comparable with or significantly exceeding (for sensitivity and kappa) the values obtained for the three-item fixed-length assessment, but not the five-item version. Finally, JITA-EMA yielded 81% sensitivity, 99% specificity, and kappa = .78, comparable with or significantly exceeding (for sensitivity) the values for the five-item fixed-length assessment (see Table 1).

**Fig. 3:**
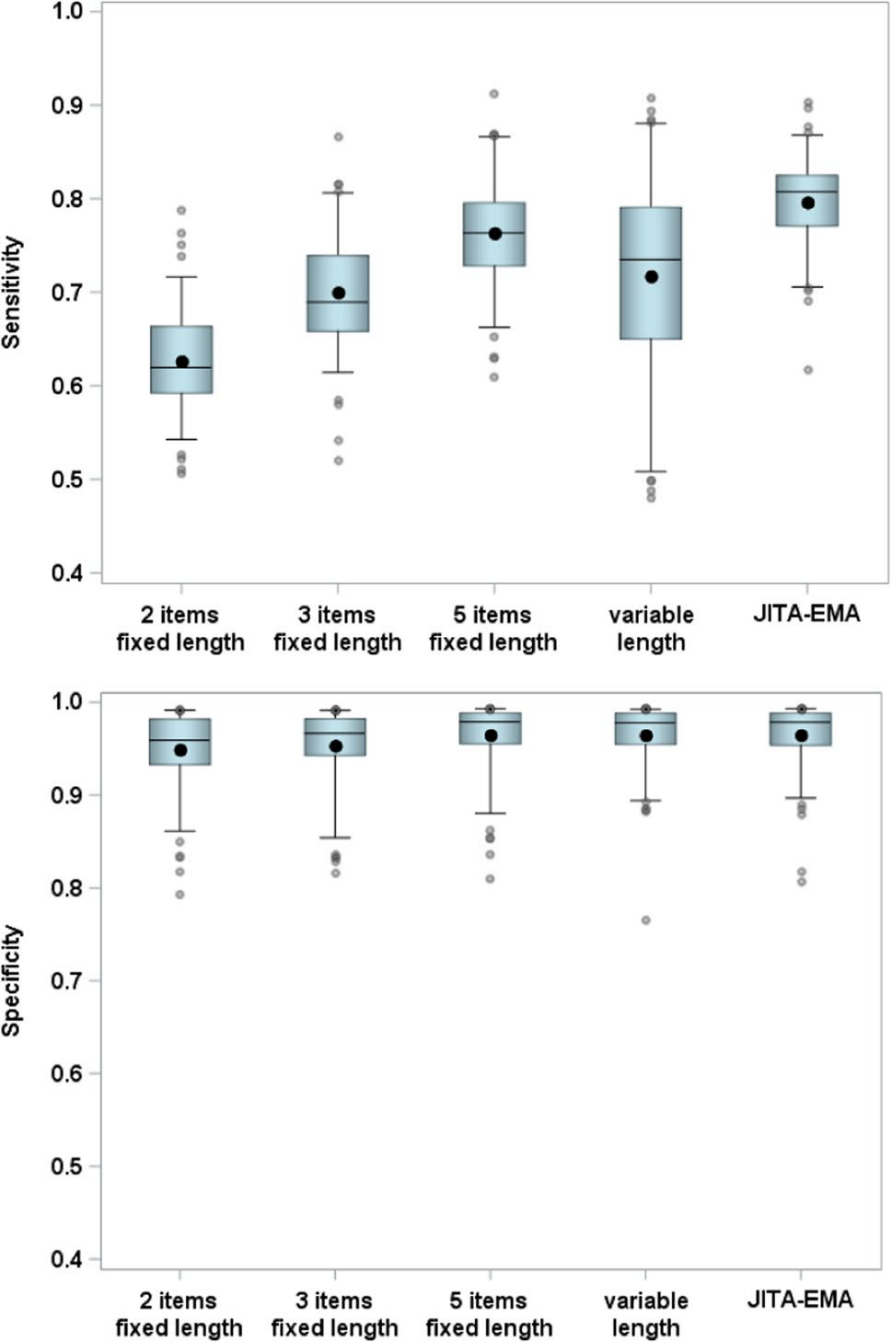
Box-and-whisker plots of individual-specific classification sensitivities (top) and specificities (bottom) across the five conditions in study 1. Black dots represent the means of the estimated sensitivities and specificities. The horizontal line in each box represents the median, the length of each box represents the interquartile range, whiskers represent the 5th and 95th percentiles, and circles represent values below the 5th and above the 95th percentile

To compare the magnitude of *individual differences* in classification accuracies across EMA item selection methods, we estimated individual-specific sensitivities and specificities from the logistic random effects models and plotted their distributions for each method. As shown in Fig. 3, the between-person variance of sensitivities was comparable for the two-item (interquartile range, IQR = 59–66%), three-item (IQR = 66–74%), five-item (IQR = 73–80%), and JITA-EMA (IQR = 77–83%) methods. A wider between-person variance was evident for the specificities when using the variable-length method (IQR = 65–79%); this suggests that, while the variable-length method had higher specificity rates than two-item fixed-length EMA on average, it yielded a wider dispersion of sensitivities across persons than other methods. The between-person variation in specificities was similar across methods (see Fig. 3).

**Fig. 4:**
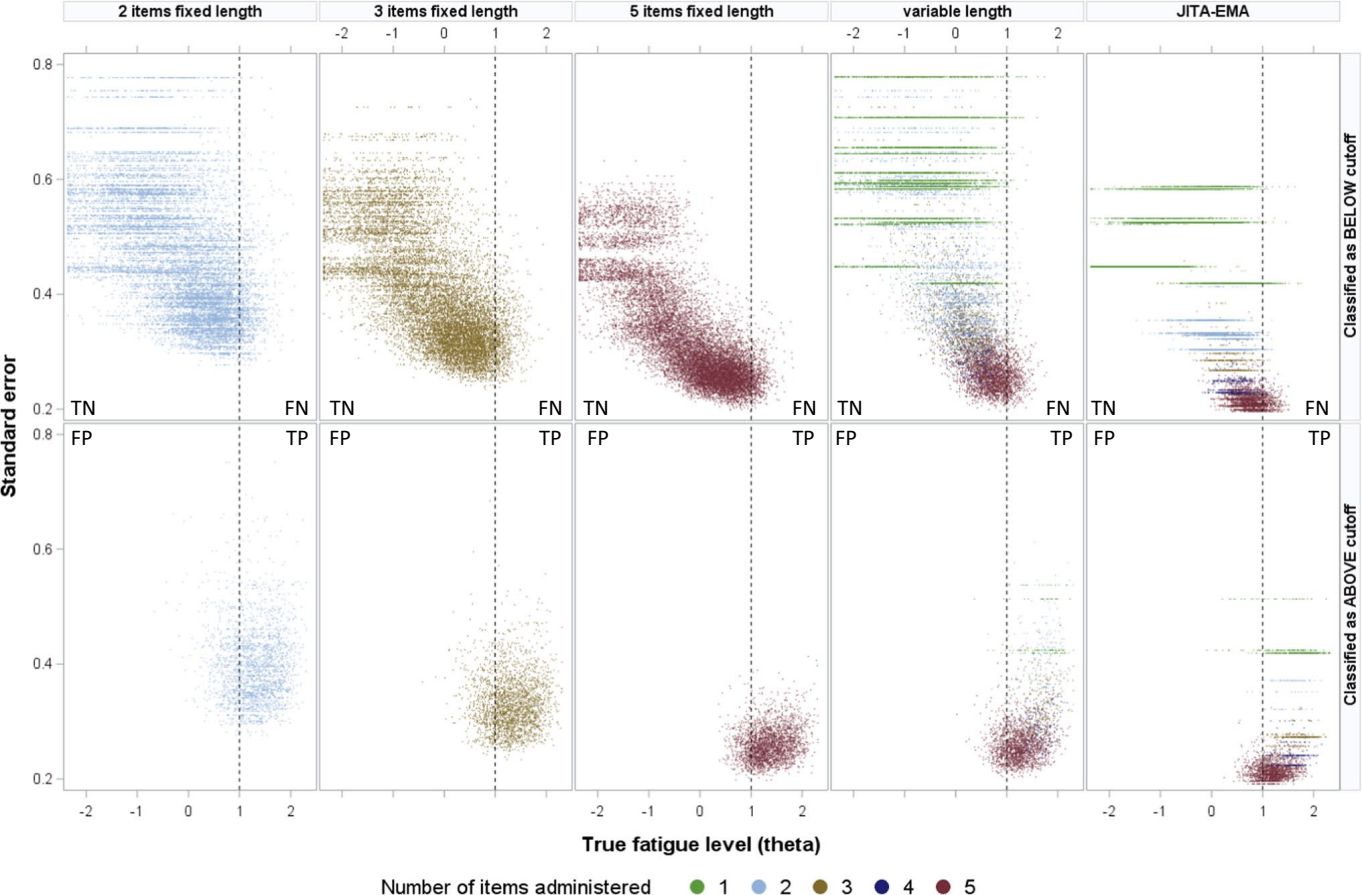
Standard errors of measurement by true fatigue scores for different numbers of items administered in each of the five conditions of study 1. TN = true negative, FN = false negative, FP = false positive, TP = true positive. True fatigue levels smaller than 1.0 (left of the dotted vertical lines) are truly below the cutoff, and true fatigue levels at or exceeding 1.0 (right of the dotted vertical lines) are truly above the cutoff

To illustrate the differences in the performance of the item selection methods, we graphically inspected the reliabilities (the standard errors of measurement, SEM) of momentary fatigue assessments for each method. Figure 4 shows the SEM (*y*-axis) plotted against the intensity levels of the true momentary fatigue scores (*x*-axis) by classification status, where the coloring of the dots indicates the number of items administered at the EMA prompt. As would be expected, increasing the number of items using fixed-length methods with two, three, or five EMA items reduced the SEM (i.e., increased the reliability of assessments) across the full range of true fatigue intensity levels, including those moments at which fatigue was far from the cutoff and at which precise assessment is unnecessary for accurate classification. The variable-length method successfully administered only one or two items at those moments at which fatigue levels were not near the cutoff, with a wide spread of SEMs across prompts. Compared to the variable length method, JITA-EMA yielded smaller SEMs for any given number of items administered, suggesting that it was successful at adaptively selecting those items that most efficiently reduced the SEM.

### Study 2 results

In this study using dynamically tailored cutoffs, the efficiency of momentary data collection may not only differ across item selection methods, but it may also change over time as more information is accumulated about each individual’s dynamic cutoff values over the course of a study. For this reason, we examined the performance of the EMA item selection methods overall and separately for each week (i.e., weeks 1–4) of the study.

#### Number of items administered

On average across all 4 weeks, the variable-length method administered a mean of 3.41 items per prompt, and JITA-EMA a mean of 2.98 items (see Table 2; the difference between the two methods was significant at *p* < .01), with minimal differences across weeks (variable-length: range = 3.37 to 3.42 items; JITA-EMA: range = 2.97 to 3.00 items). For both methods, the number of items administered showed much more pronounced within-person variation (*SD* = 1.42 for variable-length, *SD* = 1.43 for JITA-EMA) compared to the between-person variation (*SD* = .42 for variable-length, *SD* = .48 for JITA-EMA); the within-person variation accounted for 92% (variable-length) and 90% (JITA-EMA) of the total variance. Thus, even though some respondents received on average more EMA items to complete than others, the number of items delivered differed mostly across prompts within individuals with the cutoffs being tailored to each individual in this study.

**Table 2:** Mean number of items administered, sensitivity, specificity, and kappa by EMA item selection method in study 2

	Mean number of items per prompt	Sensitivity	Specificity	Kappa
Two items fixed	2.00	.68 [.65;.72] ^a^	.86 [.84;.88] ^a^	.54 [.50;.58] ^a^
Three items fixed	3.00	.72 [.69;.75] ^b^	.87 [.84;.89] ^a^	.58 [.54;.62] ^b^
Five items fixed	5.00	.78 [.75;.81] ^c^	.88 [.86;.90] ^b^	.66 [.62;.69] ^c^
Variable length	3.41 [3.29; 3.52]	.75 [.72;.78] ^b^	.88 [.86;.90] ^b^	.63 [.59;.66] ^c^
JITA-EMA	2.98 [2.86; 3.10]	.80 [.77;.83] ^c^	.90 [.88;.92] ^c^	.70 [.66;.73] ^d^

*Note*. Values in squared brackets are 95% credible intervals. Sensitivity, specificity, and kappa values with different superscripts are significantly different between EMA item selection methods at *p* < .05

#### Classification accuracy

As shown in Table 2, overall across all study weeks, administering more items using the various fixed-length methods yielded median sensitivities ranging from 68% (2 items) to 78% (5 items), specificities ranging from 86% (2 items) to 88% (5 items), and kappas ranging from .54 (2 items) to .66 (5 items). The variable-length method showed accuracies in between those of the three- and five-item fixed-length methods. JITA-EMA yielded the highest classification accuracy, with 80% sensitivity (significantly exceeding all other methods except five-items fixed-length), 90% specificity (significantly exceeding all other methods), and kappa = .70 (significantly exceeding all other methods).

**Fig. 5:**
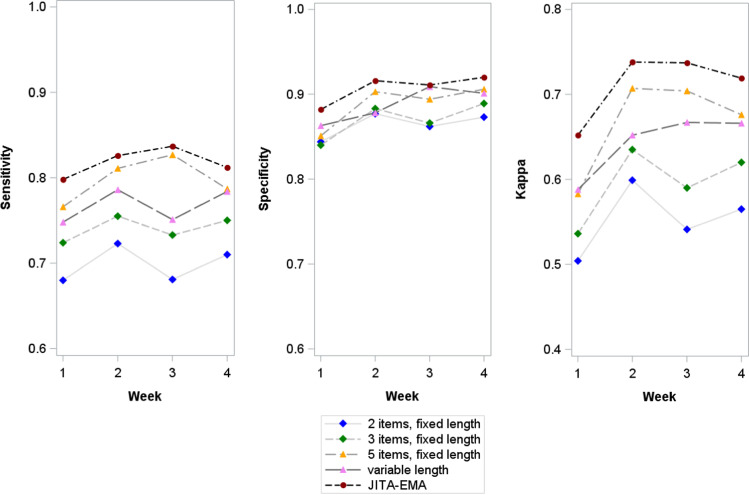
Sensitivity, specificity, and kappa by week and EMA item selection method in study 2

Examining the classification accuracies for each of the 4 study weeks, we found that the sensitivities, specificities, and kappa values were generally lower for week 1 compared to the subsequent weeks (see Fig. 5). This pattern was similar for all EMA item selection methods. Despite only administering 2.97 to 3.00 items per prompt, however, JITA-EMA consistently performed as well or better than a five-item fixed-length assessment at each week.

#### Supplemental analyses: precision of estimated dynamic cutoff values

**Fig. 6:**
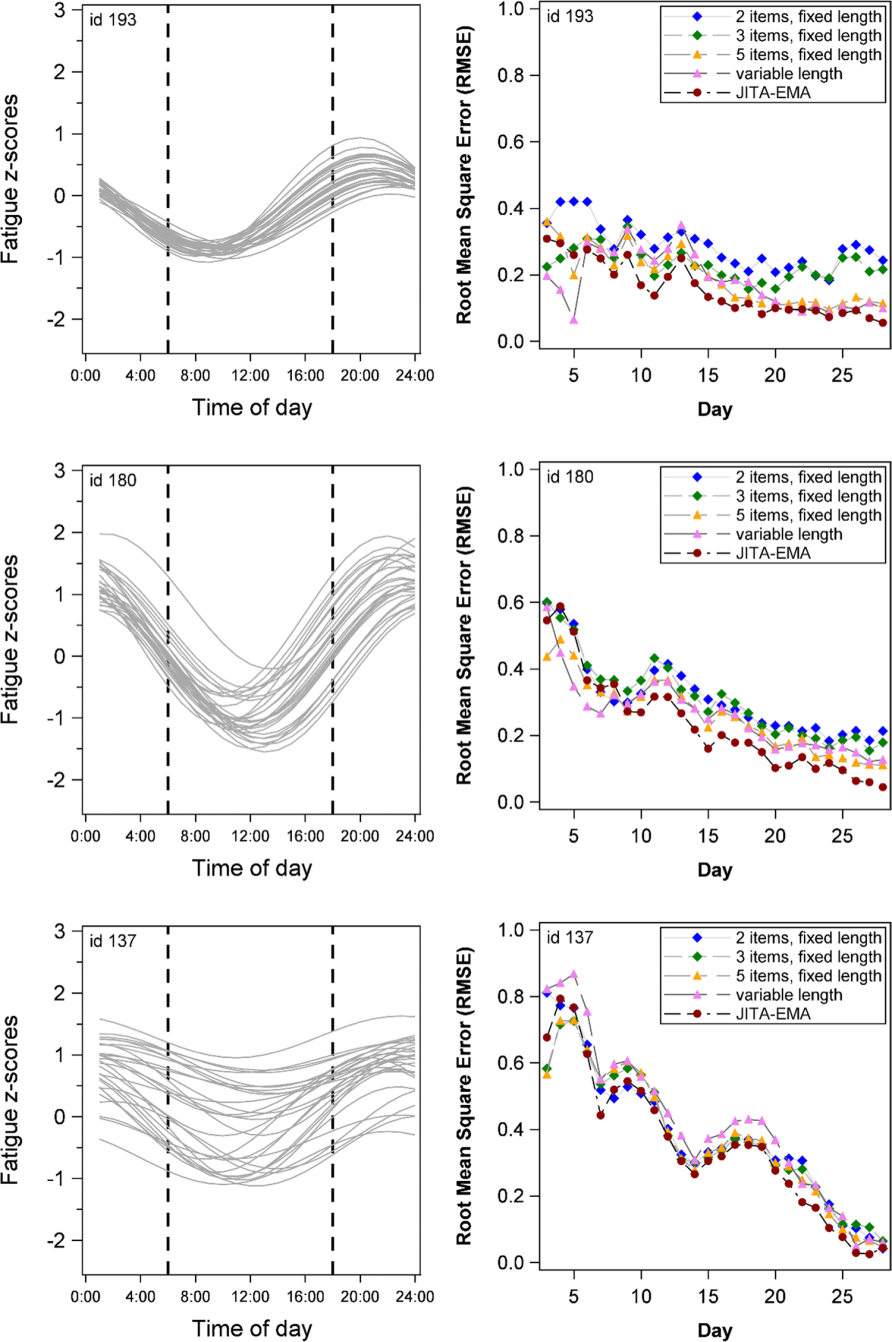
Daily diurnal cycle (left panel) and root mean square error of observed classification cutoffs by study day (right panel) for three selected individuals, study 2

As explained above, the dynamic cutoff values (i.e., ideographic fatigue diurnal cycles) used for classification were estimated every time a person received a new EMA prompt based on the observed fatigue scores obtained over the course of the study up to the current prompt, such that the dynamic classification cutoff itself was estimated with error due to uncertainty about a person’s true fatigue diurnal cycle. This error can be expected to be greater for individuals with fatigue diurnal cycles that vary markedly from day to day compared to individuals with more consistent fatigue cycles. To illustrate this, the left panel of Fig. 6 shows the day-to-day variation in diurnal cycles for three selected individuals in the sample (each gray line represents the diurnal cycle for a day, derived from a multilevel cosinor model in which true fatigue scores were nested in study days). The diurnal pattern is most consistent across days for the first person (ID 193), moderately consistent for the second person (ID 180), and least consistent for the third person (ID 137). The right panel of Fig. 6 shows the root mean square errors (RMSEs) of the observed dynamic classification cutoffs for the three individuals per study day and for each EMA item selection method. Lower RMSEs indicate less error in the observed cutoffs. As expected, the RMSEs are lowest for the most consistent person (ID 193) and highest for the person with the least consistent diurnal cycle (ID 137). The RMSEs decrease over the course of the study as more EMA fatigue data to estimate the dynamic classification cutoff are sampled and the uncertainty about an individual’s cutoff decreases.

**Fig. 7:**
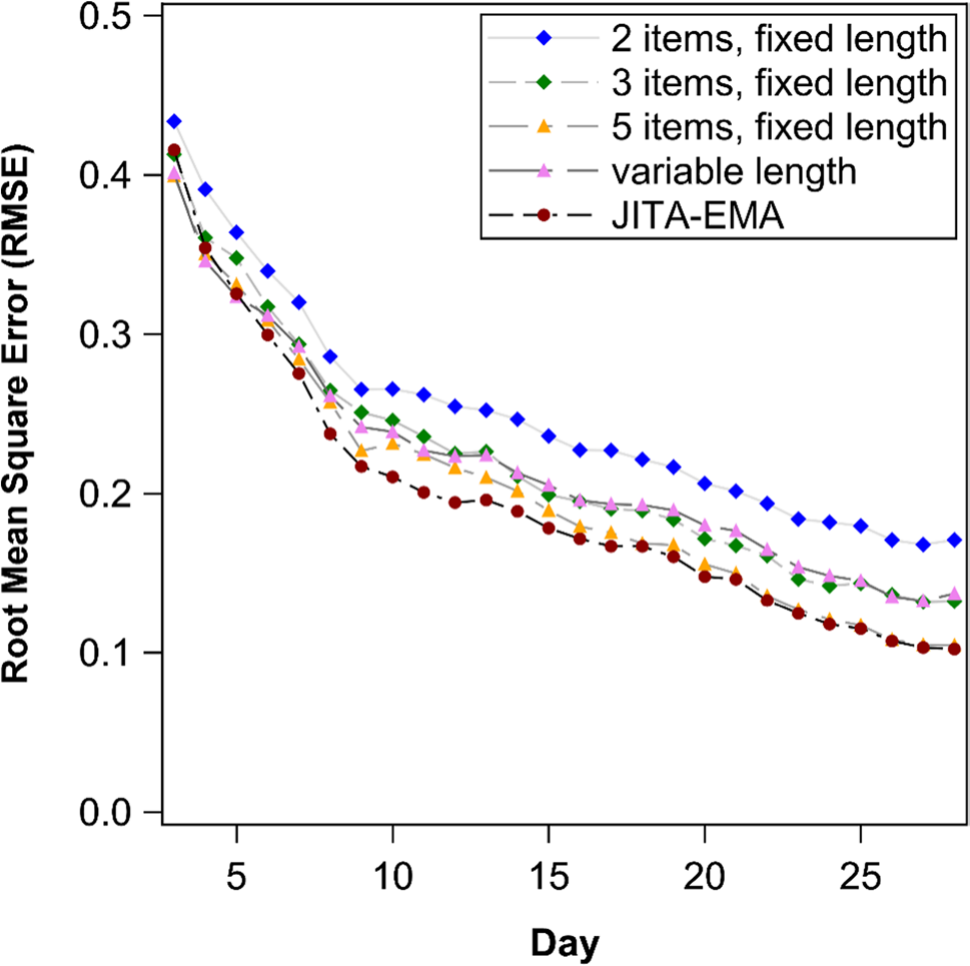
Root mean square error of observed classification cutoffs across all individuals by day in study 2

Importantly, besides sampling more time points, the uncertainty in the observed classification cutoffs may also be partially impacted by measurement error in the persons’ fatigue levels obtained at each EMA prompt, where the amount of measurement error differs between the EMA item selection methods. Figure 7 shows the RMSEs of the observed classification cutoffs across all individuals for each EMA item selection method. The RMSEs decreased similarly across study days for each of the methods, but they were consistently the highest when using a two-item fixed-length method (for which the amount of measurement error in each individual EMA prompt was the highest), and the lowest when JITA-EMA was used. This suggests that the higher classification accuracy of JITA-EMA can in part be attributed to lower errors in correctly identifying the dynamic classification cutoff values.

## Discussion

In this article, we proposed JITA-EMA as a method to enable more efficient and accurate decisions about putative moments of risk or opportunity to guide the timing of treatment delivery in JITAI when these decisions are based on EMA. It is widely recognized that measurement error can undermine and even nullify any attempt to deliver interventions at the right moments in time (Carpenter et al., 2020; Collins et al., 2004). To date, however, research on the amount of measurement error in EMA is surprisingly rare (Calamia, 2019; Cranford et al., 2006; Scott et al., 2018), and there has not been any research on the extent to which this error impedes accurate categorizations of an individual’s current state. As would be expected, our simulations showed that measurement error in EMA directly translates into inaccurate classifications of a person’s momentary states. We purposefully designed our simulations using actual data to have a realistic starting point and applying a fatigue instrument with established high reliability (Lai et al., 2003). In this context, assessing momentary fatigue with two items using a traditional fixed-length strategy resulted in considerable false negative rates where 32% (study 2) to 37% (study 1) of moments that were truly above the cutoff for “elevated fatigue” remained undetected (i.e., sensitivities of .68 and .63, respectively). Classification accuracy improved as more (3 or 5) items were administered using fixed-length assessments, consistent with typical reliability gains associated with longer measurement instruments. Our results suggested that compared to these fixed-length assessments, the efficiency of EMA can be improved with a variable-length stopping rule and, even more so, when using adaptive item selection with JITA-EMA. In both studies, JITA-EMA resulted in classification accuracies comparable with or better than using five items per prompt while reducing the number of items by 54% (study 1) and 40% (study 2) compared to a fixed-length assessment with five items. This suggests that researchers could increase (potentially nearly double) the number of constructs assessed with EMA without increasing participant burden and without compromising classification accuracy. JITA-EMA also helped with estimating more exact cutoff values when we tested a dynamic classification scenario in study 2, suggesting that the techniques may more broadly benefit the ability to make more precise momentary classifications that may inform treatment decisions.

### Considerations for JITA-EMA implementation

As we have illustrated throughout the paper, applying the adaptive assessment technique underlying JITA-EMA comes with much flexibility and many intricate decisions, many of which require conceptual considerations for which there currently is only very limited guidance available. Our goal in the presented simulations was not to identify and recommend the single best-performing version of JITA-EMA, but to introduce the technique and to illustrate the decisions that go into its implementation. In our simulations, we programmed the CAT algorithm in JITA-EMA to initialize the selection of items based on the result from the prior EMA prompt (study 1) or based on the estimated fatigue diurnal cycle of an individual (study 2). In other settings, there will undoubtedly be alternative and additional sources of information (e.g., background demographic characteristics, information about current location and activities) to initialize the best selection of items. We further allowed JITA-EMA to administer a maximum of five items from one specific item bank (the FACIT-F), applied one out of many available CAT item selection criteria (the Maximum Expected Information criterion; Choi & Swartz, 2009), and asked JITA-EMA to stop the assessment as soon as there was 95% confidence (arbitrarily selected) about a classification decision. For each of these choices there are many plausible alternatives. The dynamically tailored cutoff in our second study was further tailored to the individuals’ expected diurnal rhythms, predicted from each person’s estimated fatigue scores after an arguably brief run-in period. Because our data were not from an intervention, these diurnal rhythms were assumed to remain unchanged over time, whereas they may be expected to change over the course of an intervention, and one could attempt to adjust the classification cutoffs accordingly (e.g., by adding linear or nonlinear temporal trends to the regression model used for selection of the cutoff, assuming that the functional form of the expected temporal trends can be a priori defined). All of these factors could potentially impact the accuracy and efficiency of JITA-EMA in unknown ways. Nevertheless, as illustrated in this paper, CAT simulations (e.g., using mirtCAT) provide a powerful tool to estimate the effect of different versions of adaptive (and nonadaptive) item selection, and may aid researchers who wish to make more informed decisions about how to configure JITA-EMA (or how to choose among different fixed-length EMA surveys) in their studies.

### Considerations for EMA item banks

Given that JITA-EMA is based on adaptive item selection from a larger bank of items, its functionality squarely rests on the availability of psychometrically sound item banks that meet the assumptions of IRT. In the last few decades, calibrated item banks have been developed for a large and increasing number of self-reported symptoms, health behaviors, and emotional experiences (e.g., Cella et al., 2010; Gershon et al., 2012; Tulsky & Kisala, 2015), but most existing item banks were developed for use in traditional (e.g., cross-sectional) settings and use self-reports with longer (e.g., past 7 days) recall periods. In the present simulation studies, we used the FACIT-F, assuming that the instrument could plausibly serve as an item bank for momentary assessments. It is customary in EMA research to adapt existing instruments by modifying the recall period to fit with momentary self-reporting, but the extent to which this modification impacts the psychometric characteristics of self-report instruments is not well understood (Schneider et al., 2013). Importantly, for a set of items to be suitable for use with CAT and CCT, they need to perform in the same way across different groups or time points (i.e., show no “differential item functioning,” DIF). This requirement is especially challenging when it comes to the measurement of dynamic changes within individuals with EMA, because items can function differentially (show DIF) across within-person contexts (e.g., location, time of day) and may gradually shift their meaning over time and with repeated assessments (item parameter “drift”) (Schneider & Stone, 2016). Such within-person DIF effects may even differ across groups or individuals. These complexities notwithstanding, paying close attention to the importance of psychometrically sound momentary instruments would not only benefit the implementation of JITA-EMA, but also the standardization, replicability, and transparency of EMA measures in general.

## Limitations and directions for future research

The present study has several limitations. We used data from a real-world example to evaluate the performance of JITA-EMA and other item selection methods. Even though this allowed us to simulate the momentary classifications a researcher would have obtained in an actual study, our results are limited to a single data set and a sample of patients with chronic pain, and the results do not necessarily generalize to other samples or conditions.

Furthermore, the simulations were based on data from an observational (not a JITAI) study in which participants showed high compliance (91% on average) with the EMA protocol, whereas EMA completion rates are typically lower in observational EMA studies (May et al., 2018; Wen et al., 2017) and in JITAIs using EMA (Perski et al., 2022). Even though missed EMA prompts will not affect the CAT item selection process at a given EMA prompt, they may affect the quality of information available about an individual when initial item selection and classification cutoffs are dynamically tailored to scores from previous EMA prompts. Especially when data are missing not at random (e.g., when EMA prompts were more likely missed when fatigue is high), systematic bias may be introduced into the estimation of dynamically tailored cutoff values. The impact of different missing value patterns on the accuracy and efficiency of EMA item selection methods (including JITA-EMA) could be examined in future simulations.

Our simulations were based on a single treatment tailoring variable, fatigue. Although this was deemed reasonable for the present proof-of-concept demonstration, treatment decisions in JITAI are not necessarily based on only one variable. Even though the majority of JITAIs utilize EMA for treatment decisions (Perski et al., 2022), they often integrate input from multiple EMA measures or combine EMA with passively collected (e.g., geolocation) data (Hardeman et al., 2019; Perski et al., 2022). Techniques to combine the adaptive administration of items across multiple self-report measures using multivariate CAT are already available (Morris et al., 2017). The mirtCAT package is suitable for simulation and real-time application of both univariate and multivariate computerized adaptive tests (Chalmers, 2016). In a multivariate CAT, information from correlated latent constructs is leveraged to increase efficiency, such that those items are selected to help most quickly increase the information (i.e., reduce the measurement error) for two or more latent constructs simultaneously. JITA-EMA applications could be expanded accordingly, keeping in mind that this increases the number and complexity of CAT and CCT design choices (e.g., whether the selection of items should prioritize the reduction of measurement error for constructs that are deemed more important for classification than others, and whether stopping rules to terminate the assessment should be applied globally or specifically for each construct).

We have presented and discussed JITA-EMA with a focus on precision and efficiency of measurement, but researchers may also want to control the content of EMA items selected. An obvious concern is that when respondents receive the same items dozens of times, they may start to engage in survey satisficing and careless responding (Jaso et al., in press). This issue is inherent in conventional EMA that administers the same set of items at every prompt (Silvia et al., 2014), but it can also arise in CAT where items that are overall more informative (i.e., those that tend to most quickly reduce measurement error) may be administered more frequently than overall less informative items. CAT item selection methods can be adjusted by including “exposure control” strategies to help reduce excessive use of highly informative items, or by including content balancing strategies to ensure that various contents covered by the items in a bank are covered at each assessment occasion (Chalmers, 2016; Leung et al., 2003; Stocking & Lewis, 2002). Correspondingly, JITA-EMA could be coupled with exposure control and/or content balancing methods to avoid repeated selection of the same items over multiple EMA prompts. Although this might possibly enhance participant engagement and facilitate careful responding, it should also be noted that, at present, there is no clear evidence to support this conjecture. It is also possible that frequently switching the specific items (including their order) across prompts could confuse respondents, require more time, and lead to frustration, disengagement, and lower compliance with completing assessments. An advantage of CAT item selection methods is that different degrees of exposure control and content balancing can be directly manipulated, and this feature could be leveraged in future research to experimentally compare the extent to which higher versus lower redundancies in the selection of items across prompts affects participant behavior and the quality of EMA data.

A critical component of treatment tailoring in JITAI is deciding not only when to deliver an intervention, but also which type, components, and dosage should be delivered at a given point in time (Nahum-Shani et al., 2018). These decisions are not within the reach of JITA-EMA. The technique is intended only to help improve the detection of momentary states of risk or opportunity by providing more efficient classifications of momentary states. A possible direction for future work is to integrate JITA-EMA into larger control system engineering models (e.g., Deshpande et al., 2014) that aim to translate real-time observations into adaptive treatment decisions.

Finally, an important direction for future work would be to test the effects of JITA-EMA in actual studies. This includes not only effects on item delivery (average number and distribution of items, time to complete each survey), but also whether JITA-EMA might affect EMA data quality by impacting EMA completion rates, reducing perceived assessment burden, and minimizing potential careless responses. Ultimately, what matters most and can only be addressed in a real study is whether JITA-EMA has the potential to improve JITAIs, including potential effects on sustained participant engagement and satisfaction with the intervention, and, most importantly, intervention efficacy.

## Conclusion

Random error in measurements directly translates to unsystematic and “noisy” tailoring decisions in JITAIs (Collins et al., 2004), and participant burden associated with extensive momentary self-reports may demotivate participants engaged in these interventions (Goldstein et al., 2020). JITA-EMA represents one approach to address these two issues with adaptive item selection applied to EMA. Employing this tool in future research could set the stage for a more optimal implementation of EMA in JITAIs.

## Footnotes

1 For the fixed-length methods, instead of administering a random subset of items, we could have (arbitrarily) selected the same subset of items from the 13-item FACIT-F to be administered at each prompt. Even though this would more closely mirror current practice of EMA measurement, the results would have been influenced by the specific subset of items chosen. Randomly selecting a subset of items approximates the expected average performance of traditional fixed-form EMA strategies in which any given subset of two, three, or five items could have been selected for administration in an EMA study (see Silvia et al., 2014).

2 We do not suggest that a “run-in” period of only 2 days would be considered ideal in most real applications. We selected 2 days as the minimum run-in period after which it was possible to fit a cosinor regression model to the fatigue scores observed for each person at the previous prompts (and to estimate dynamic classification cutoffs for the purposes of the simulation).

3 Kappa can be expressed as κ = [2*Pr*(1–*Pr*)(*Se*+*Sp*–1)]/[*Pr*^2^+(1– *Pr*)^2^+(1–2*Pr*)(*PrSe*–(1–*Pr*)*Sp*)], where *Se* represents sensitivity, *Sp* represents specificity, and *Pr* represents prevalence (i.e., the probability of moments being truly above the cutoff). We applied this formula using the MODEL CONSTRAINTS feature implemented in M*plus* software to derive kappa (with 95% credible intervals) from the estimated between-person intercepts of logit sensitivity, specificity, and prevalence in logistic multilevel models with linear parameter constraints. This also allowed for comparisons of (correlated) kappas across EMA item selection methods based on multivariate logistic multilevel models.

4 The specificity (true negative rate) of classifications is naturally high in study 1 given the high classification cutoff of 1 *z*-score above the mean fatigue value results in a high true negative rate.
